# A case of solitary plasmacytoma of bone showing co-expression of both immunoglobulin light chains

**DOI:** 10.1186/s40001-021-00621-8

**Published:** 2021-12-20

**Authors:** Ryota Matsuoka, Noriaki Sakamoto, Takayasu Kato, Shigeru Chiba, Masayuki Noguchi

**Affiliations:** 1 Department of Diagnostic Pathology, Faculty of Medicine, University of Tsukuba, 1-1-1 Tennodai, Tsukuba, Ibaraki 305-8577 Japan; 2 Department of Hematology, Faculty of Medicine, University of Tsukuba, 1-1-1 Tennodai, Tsukuba, Ibaraki 305-8577 Japan

**Keywords:** Solitary plasmacytoma of bone, Dual immunoglobulin light chain expression, Allelic exclusion, Isotypic exclusion, Case report

## Abstract

**Background:**

Solitary plasmacytoma of bone (SPB) is a rare plasma cell neoplasm. It arises in bone as a single locus in the absence of any plasma cell myeloma lesions. Plasma cell neoplasms intrinsically express only one immunoglobulin light chain (IgL)—kappa or lambda—and using this fact, kappa/lambda deviation is the decisive factor for diagnosis. Co-expression of both IgLs in a single tumor cell is extremely rare.

**Case presentation:**

We report a case of SPB that arose in the vertebra of a 52-year-old Japanese woman. Histologically, the resected mass showed diffuse plasma cell proliferation. Dual IgL expression was detected by flow cytometry, immunohistochemistry, and in situ hybridization (ISH) targeting IgL mRNA.

**Conclusion:**

We have presented an extremely rare case of SPB showing dual expression of kappa and lambda IgLs. This unusual case of plasma cell neoplasia might represent a possible exceptional example of failure of “IgL isotypic exclusion.”

**Supplementary Information:**

The online version contains supplementary material available at 10.1186/s40001-021-00621-8.

## Background

Solitary plasmacytoma of bone (SPB) is a rare subtype of plasma cell neoplasm, accounting for less than 5% of plasma cell neoplasms overall. SPB may develop at any bone site, but is especially associated with active bone marrow hematopoiesis. Reported sites of origin have included the vertebrae, ribs, and pelvis [1, 2]. Tumor cells resemble plasma cells which have abundant basophilic cytoplasm, often with pale paranuclear area. The nuclei are located eccentrically and contain condensed chromatin. Tumor cells are positive for cytoplasmic immunoglobulin (Ig), surface Ig is usually undetectable, and M-proteins are often present, with IgG being the most common type (about 60%), IgA (15%), and IgE and IgD (about 1%) [3]. Tumor cells are usually negative for CD45 and the pan-B-cell markers CD19 and CD20, and positive for CD38, CD79a, and CD138. Cytologically and immunophenotypically, SPB is similar to plasma cell myeloma (PCM), although it presents as a single localized neoplasm, shows no evidence of bone marrow involvement, and no clinical or laboratory evidence of PCM is evident in affected patients. The median age of patients with SPB is 55 years, i.e., about 10 years younger than those with PCM, and males are affected twice as frequently as females. SPB is treated mainly with radiation and surgery, with additional chemotherapy in cases that have progressed to PCM. The prognosis of SPB is better than that of PCM, and 50% of SPB patients survive longer than 10 years. More than 50% of SPB cases progress to PCM within 2 years or less, suggesting that some cases of SPB represent an early manifestation of PCM [1, 4–6].

A number of cytokines have been shown to be involved in the pathogenesis of plasma cell neoplasm [7, 8]. These factors, secreted by tumor cells or the surrounding microenvironment, have been implicated in tumor growth and apoptosis, and may also result in the secretion of pathological Ig. It has also been suggested that these cytokines may affect the immune status of plasma cell neoplasm patients [9, 10].

Normal B cells or plasma cells express a single Ig kappa or lambda light chain, and the ratio of B cells or plasma cells expressing kappa relative to those expressing lambda ranges from 0.5 to 3.0 [11, 12]. B cell and plasma cell neoplasms are believed to arise from a single cell after initiation of immunoglobulin heavy chain (IgH) and immunoglobulin light chain (IgL) rearrangement, and the neoplastic plasma cells essentially express a single type of IgL. Thus, restriction of IgL expression can be detected by flow cytometry, in situ hybridization and immunohistochemistry. In neoplastic plasma cells, co-expression of both IgL types is extremely rare. Here, we report a very rare case of SPB showing dual expression of IgL.

## Case presentation

A 36-year-old woman presented to a local hospital with a history of neck pain. Computed tomography (CT) and magnetic resonance imaging (MRI) demonstrated a tumor arising from the anterior elements of the C1 and C2 vertebrae. Cervical spine fusion and mass reduction surgery were performed. The resected specimen showed diffuse proliferation of plasma cells and was diagnosed as “plasmacytoma” at that time. Additional radiotherapy was performed, but the patient later dropped out from the treatment course.

**Fig. 1:**
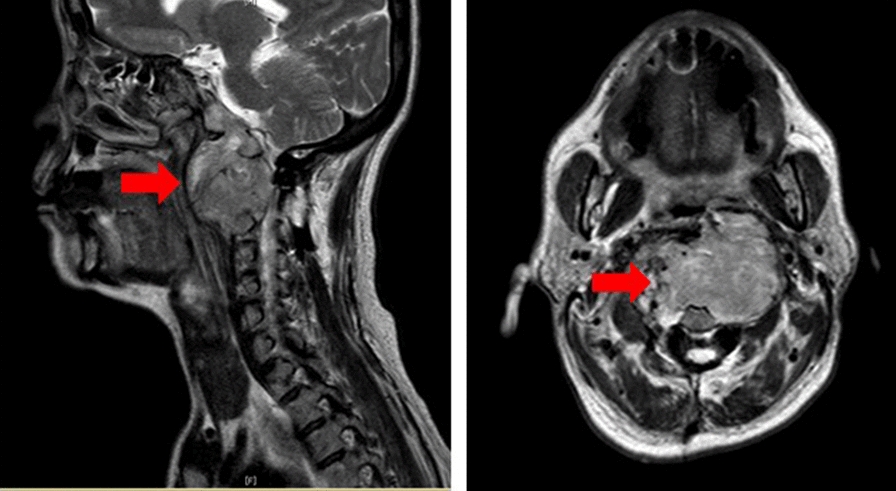
T2-weighted MRI revealed a hyper-intensity signal mass lesion extending from the clivus to the atlas vertebra (red arrow)

Sixteen years later, at the age of 52 years, the patient returned complaining of dysarthria. CT and MRI showed a similar but much larger mass at the same location, and recurrence of the tumor was diagnosed (Fig. 1). The mass compressed the spinal cord and was thought to be responsible for the dysarthria. The patient was referred to our hospital for further examination and treatment. Quantitative serum Ig analysis showed an increased level of IgG (2096 mg/dl; reference range 870–1700 mg/dl) and normal levels of IgM (203 mg/dl; reference range 46–260 mg/dl) and IgA (293 mg/dl; reference range 110–410 mg/dl). A serum Ig-free light chain study revealed increased levels of both free kappa light chain (61.5 mg/l; reference range 2.42–18.92 mg/l) and free lambda light chain (88.1 mg/l; reference range 4.44–26.18 mg/l) (kappa/lambda: 0.70).

**Fig. 2:**
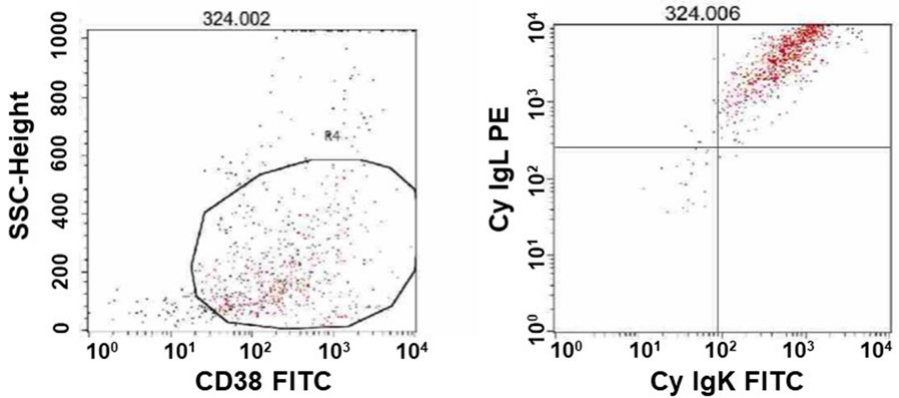
Flow cytometry of the tumor revealed that CD38-positive cells co-expressed kappa and lambda light chain. Fluorescein isothiocyanate (FITC)-labeled CD38 antibody (T16, Beckman Coulter, Inc., CA), FITC-labeled cytoplasmic kappa light chain antibody [Rabbit Polyclonal F(ab′)2, Agilent Technologies, CA], and phycoerythrin (PE)-labeled cytoplasmic lambda light chain antibody [Rabbit Polyclonal F(ab′)2, Agilent Technologies, CA] were used for the flow cytometry

**Fig. 3:**
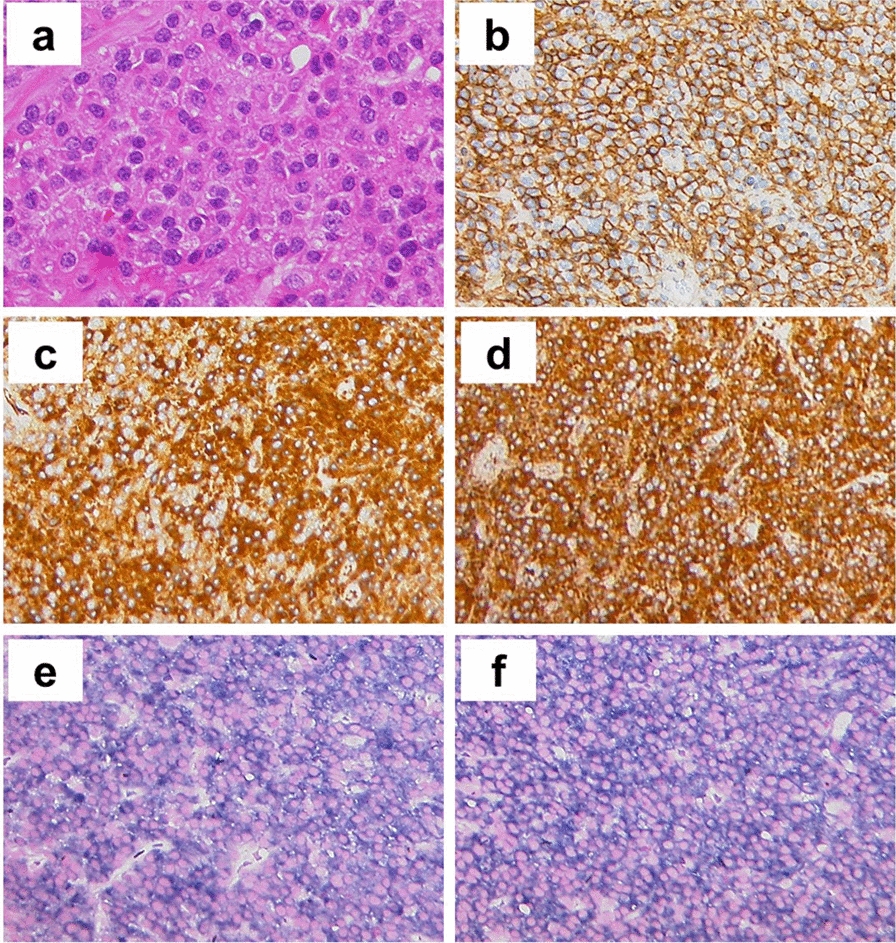
Tumor biopsy of the neck (sampled at 2019). **a** H&E staining (×400) shows marked plasma cells infiltration. **b** CD138 staining (×200) shows diffuse positivity for these plasma cells. **c**, **d** In situ hybridization of kappa and lambda light chain mRNA (×200) and **e**, **f** immunohistochemistry of kappa and lambda light chain (×200)

Excisional biopsy of the tumor was performed. Flow cytometry analysis demonstrated a distinct population of abnormal plasma cells which were positive for CD56 (96%), CD38 (70%), CD45 (9%), and CD19 (1%). Approximately 96% of these tumor cells co-expressed cytoplasmic kappa and lambda light chain based on a CD38-positive gate strategy (Fig. 2). Histologically, the specimen showed diffuse proliferation of plasmacytoid tumor cells with pale paranuclear area and dense chromatin, and immunohistochemistry showed that these tumor cells were strongly and diffusely positive for CD138 (Fig. 3a, b) and MUM1, and negative for CD3, CD20, and CD56. Immunohistochemistry for IgL demonstrated co-expression of kappa and lambda light chain (Fig. 3c, d). Additionally, we demonstrated in situ hybridization (ISH) targeting IgL mRNA using FFPE specimen, and ISH also revealed that tumor cells co-expressed kappa and lambda light chain (Fig. 3e, f). Bone marrow biopsy showed no evidence of plasmacytes showing dual expression or deviation of kappa and lambda light chain, and additional CT and MRI revealed no skeletal abnormality except for the primary lesion, and absence of end-organ damage attributable to a proliferative plasma cell disorder.

We reviewed the previous specimen and performed additional IgL immunohistochemistry. The sample revealed a histological pattern similar to that of the later sample as well as co-expression of kappa and lambda light chain (Additional file 1: Fig. S1).

Taking these findings together, the tumor was diagnosed as SPB with dual expression of the kappa and lambda light chains. After diagnosis, the patient underwent nine courses of VRD (bortezomib, lenalidomide, and dexamethasone) therapy, which reduced the size of the lesion. Two years after diagnosis, the lesions have not increased in size, and bone marrow examinations have shown no progression to PCM.

## Discussion

To our knowledge, this case of SPB showing dual expression of the kappa and lambda light chains is the first of its kind to have been reported. Normal plasma cells differentiate from B cells and produce Ig. B cells can rearrange their Ig genes to recognize a huge variety of different antigens. Ig is normally composed of one heavy and one light chain. There are five types of IgH—IgM, IgG, IgA, IgD, and IgE produced by a single gene—and two types of IgL—kappa and lambda produced by two distinct genes. The *IgH*, *IgL kappa*, and *IgL lambda* gene loci are located on chromosomes 14, 2, and 22, respectively. If the *IgH* and one of the *IgL* genes are rearranged successfully, the other allele of the *IgH* and *IgL* gene is excluded to maintain the antigen specificity of the B cell, a phenomenon known as “allelic exclusion.” In this way, a single B cell expresses the rearranged *IgH* and the either *IgL* genes transcribed from only one allele each, with the other 4 alleles remaining in the germline configuration. Normal B cells first undergo *IgH* rearrangement, followed by *IgL kappa* rearrangement. If a productive *IgL kappa* rearrangement occurs, the *IgL lambda* gene never rearranges. If *IgL kappa* rearrangement is non-productive for both alleles, the *IgL kappa* locus is inactivated by deletion and *IgL lambda* rearrangement occurs [13, 14]. This mechanism of expressing either *IgL kappa* or *lambda* genes is called “isotypic exclusion,” and thus B cells (or plasma cells) express one type of light chain, but not both [15]. “Isotypic exclusion” of IgL is not fully understood, although some hypotheses have been proposed on the basis of experimental studies. Inactivation of *IgL kappa* is accomplished by recombination activating gene (RAG)-mediated joining of the non-coding recombining sequence (RS). The RS is the *IgL kappa*-deleting element in humans, located ~ 25 kilobases downstream of the constant region of *IgL kappa* (C-kappa) [16, 17]. RS recombination leads to deletion of the C-kappa exon and silencing of the *IgL kappa* allele, and it is also known that RS recombination promotes the formation of B cells which express IgL lambda chain [17–19]. However, Diaw et al. reported a mouse-origin plasmacytoma that produced both IgL kappa and lambda, and using micro-manipulation and reverse transcription polymerase chain reaction (RT-PCR) confirmed that both were expressed simultaneously in a single cell [20]. This suggests that in some plasma cell neoplasms both kappa and lambda light chains may exist in a single plasma cell. Furthermore, few cases of PCM showing dual expression of IgL have been reported. The majority of such cases have tended to show a high incidence of complex cytogenetic or fluorescence in situ hybridization (FISH) abnormalities, suggesting involvement of the light chain genes, subsequent isotypic exclusion error, and dual light chain expression [21–23]. Unfortunately, as no investigation of chromosome abnormalities was undertaken in the present case, any genetic dysfunction related to isotypic exclusion remained unclear. On the other hand, Shi et al. subjected human peripheral B cells to single-cell sequencing and found that more than one antibody was produced in some individual B cells, albeit accounting for a small proportion of the total (about 10%) [24]. This unprecedented finding appears to cast doubt on the traditional “one cell – one antibody” paradigm and suggested that dual expression of kappa and lambda light chain can occur in normal conditions and might be the origin of the dual IgL expression neoplasm. However, due to the limited number of cases, further investigation is needed to elucidate the mechanism of tumor co-expression of kappa and lambda light chains.

The findings in the present case invite speculation as to how both the kappa and lambda light chains can exist in a single neoplastic plasma cell. Two hypotheses to explain this have been suggested: (1) two types of light chain are expressed in the same antibody; (2) two types of antibodies constructed by each of the kappa and lambda light chains are present in the same plasma cell. In the present case, we detected co-expression of both IgLs in the neoplastic cells by flow cytometry, immunohistochemistry and ISH targeting IgL mRNA, but none of the results offered any suggestion about either hypothesis. There is no way to test these hypotheses at this stage, and further analysis is needed.

## Conclusion

To our knowledge, the present case of SPB showing dual IgL expression is the first of its kind to have been reported. IgL rearrangement is under strict genetic control, although the mechanism involved is still unclear. The present appears to represent an exceptional event that deviates from the traditional “isotypic exclusion” mechanism.

## Supplementary Information

**Additional file 1: Figure S1.** Tumor biopsy of the neck (sampled at 2003). (**a**) H&E staining (×400) shows marked plasma cells infiltration. (**b**) CD138 staining (×200) shows diffuse positivity for these plasma cells. (**c**, **d**) Immunohistochemistry of kappa and lambda light chain (×200).

## Data Availability

All data and material are included in this published article.

## Abbreviations

SPB: Solitary plasmacytoma of bone
PCM: Plasma cell myeloma
IgL: Immunoglobulin light chain
IgH: Immunoglobulin heavy chain
CT: Computed tomography
MRI: Magnetic resonance imaging
VRD: Bortezomib, lenalidomide, and dexamethasone
Ig: Immunoglobulin
ISH: In situ hybridization
RT-PCR: Reverse transcription polymerase chain reaction
FISH: Fluorescence in situ hybridization
