# Supplementary figures and images for: A case of solitary plasmacytoma of bone showing co-expression of both immunoglobulin light chains
Source: Eur J Med Res. 2021 Dec 20;26:148. doi: 10.1186/s40001-021-00621-8 (PMC8686560; doi:10.1186/s40001-021-00621-8)

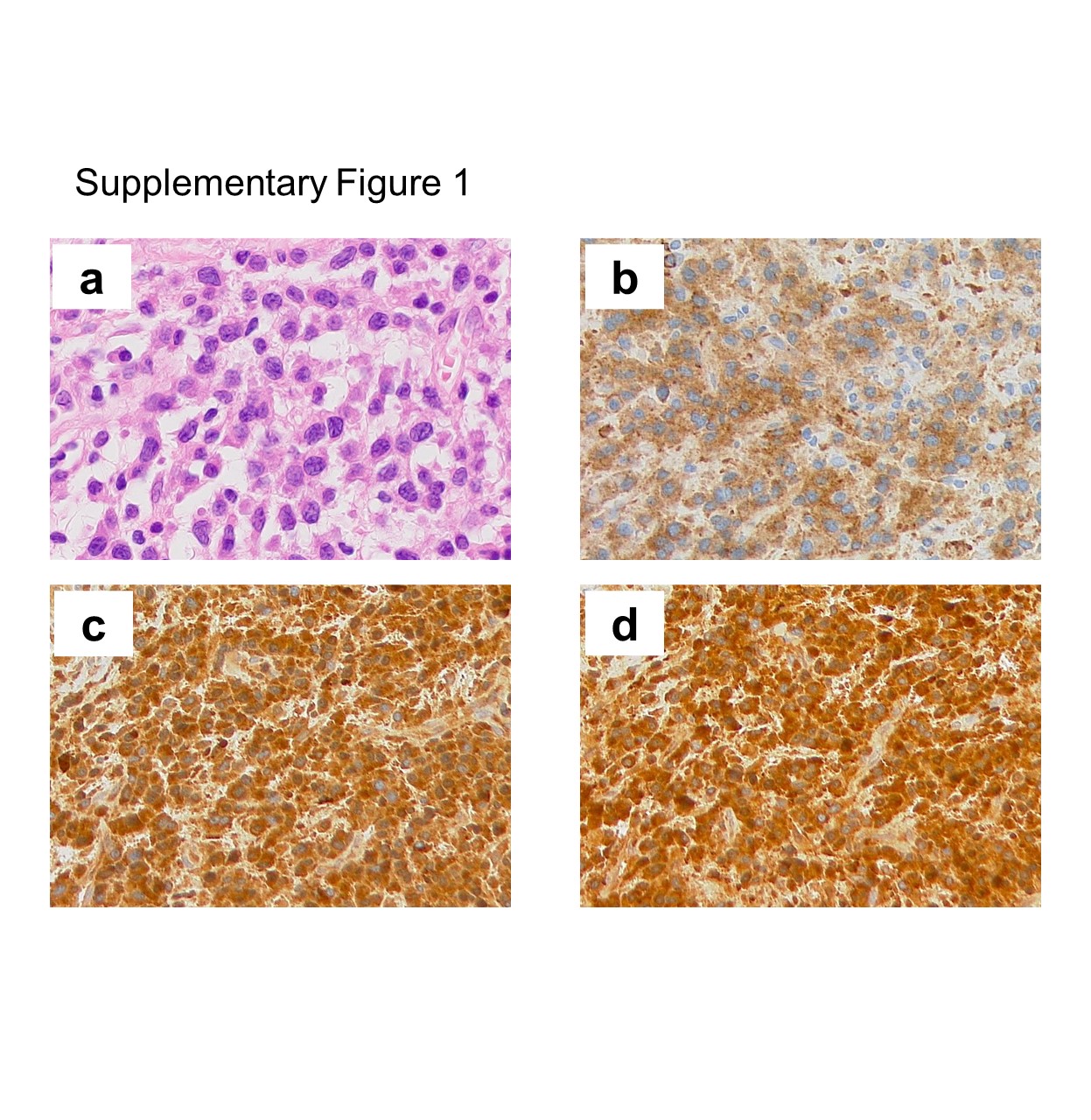

Supplement: Supplementary file 1 — Additional file 1: Figure S1. Tumor biopsy of the neck (sampled at 2003). (a) H&E staining (×400) shows marked plasma cells infiltration. (b) CD138 staining (×200) shows diffuse positivity for these plasma cells. (c, d) Immunohistochemistry of kappa and lambda light chain (×200). [file 40001_2021_621_MOESM1_ESM.jpg]
